# Using the Unified Theory of Acceptance and Use of Technology (UTAUT) to Investigate the Intention to Use Physical Activity Apps: Cross-Sectional Survey

**DOI:** 10.2196/13127

**Published:** 2019-09-10

**Authors:** Di Liu, Remina Maimaitijiang, Jing Gu, Shuyi Zhong, Mengping Zhou, Ziyue Wu, Ao Luo, Cong Lu, Yuantao Hao

**Affiliations:** 1 School of Public Health Sun Yat-sen University Guangzhou China; 2 School of Public Health The University of Hong Kong Hong Kong Special Administrative Region China; 3 Sun Yat-sen Global Health Institute Sun Yat-sen University Guangzhou China

**Keywords:** intention, physical activity apps, university students, UTAUT

## Abstract

**Background:**

Many university students are lacking adequate physical exercise and are failing to develop physical activity (PA) behaviors in China. PA app use could improve this situation.

**Objective:**

The aim of this study was to use the unified theory of acceptance and use of technology (UTAUT) to investigate the intention to use PA apps among university students in Guangzhou, China, and how body mass index (BMI) moderates the effects of UTAUT in explaining PA app use intention.

**Methods:**

A cross-sectional study was conducted among 1704 university students from different universities in Guangzhou, China. The UTAUT model was used to measure the determinants of intention to use PA apps.

**Results:**

Of the participants, 41.8% (611/1461) intended to use PA apps. All three UTAUT-related scales (performance expectancy, effort expectancy, and social influence) were positively associated with the intention to use PA apps after adjusting for background variables (adjusted odds ratio 1.10-1.31, *P*<.001). The performance expectancy scale had stronger associations with the intention to use PA apps among those whose BMI were beyond normal range compared with those whose BMI were within normal range (*P*<.001).

**Conclusions:**

UTAUT is useful for understanding university students’ intention to use PA apps. Potential moderating effects should be kept in mind when designing UTAUT-based interventions to improve PA via app use.

## Introduction

Physical inactivity is one of the biggest public health issues of the 21st century and has been identified as the fourth leading risk factor of global mortality by the World Health Organization [1,2]. Evidence has shown that regular physical activity (PA) helps balance energy, control weight, and reduce the risk of noncommunicable diseases (eg, diabetes and hypertension) and mental illness (eg, depression) [1]. Physical inactivity can lead to a worsening health condition and deterioration of quality of life [3,4]. Hallal et al [5] collected PA level data of a population aged 15 years or older worldwide and demonstrated that 31.1% were physically inactive and inactivity increased with age in all regions included. Similarly, several studies indicated an age-related decline in the level of PA throughout life and that the PA pattern in adolescence usually affected the pattern in adulthood [6-9].

Despite the importance of regular PA, many university students are now living in an environment with increased barriers to PA, resulting in a lack of adequate physical exercise and a failure to develop PA behaviors [10-13]. The 2014 National Physique Monitoring Bulletin released by the General Administration of Sport of China indicated that Chinese university student physical fitness has continued to decline [14].

Effective approaches are urgently warranted to improve this situation. Review studies showed that school-based education programs and interclass exercises could effectively promote PA and fitness among younger adolescents [15]. However, such educational programs could be less effective among university students as their PA was usually less regulated by universities. Furthermore, university students have unique characteristics (ie, they are in a transitional period between adolescence and adulthood). Therefore, innovative approaches are vital to engaging more university students in PA programs. Given the widespread use of smartphones by university students, interventions using this technology may provide a viable opportunity to reach this population and deliver interventions. One benefit of mobile health (mHealth) approaches over traditional methods is that interventions can be provided anywhere and at any time, making them potentially more accessible and feasible [16].

International Telecommunication Union reported that by 2015 Chinese people’s mobile phone ownership and internet use reached 92.18% and 50.30%, respectively [17]. University students are often early adopters of new consumer technologies such as smartphones and PA apps [18]. The number of smartphone health and fitness apps has dramatically increased in recent years, with more than 17,000 having been developed for the public [19,20]. The mHealth approaches delivered through PA apps can make PA promotion interventions more attractive and interesting [16] by incorporating strategies such as gamification [21], personalization [22], and creating social network and peer support [23]. Meanwhile, the effectiveness of using PA apps to promote PA has been examined [24,25]. A review and meta-analysis demonstrated the positive effects of PA apps on increasing PA and promoting weight loss [26]. As use behavior is directly affected by use intention according to theories in the social sciences and related domains (eg, theory of planned behavior, technology acceptance model, and unified theory of acceptance and use of technology [UTAUT]), promoting use intention before implementing interventions could facilitate intervention promotion [27-29]. Thus, assessing use intention is important.

UTAUT has been used to investigate behavioral intention to use technology and its influencing factors [30]. To the best of our knowledge, there is no published study applying UTAUT to the investigation of PA app use intention in China. As PA app use is a promising measure in health promotion, in this study, we investigated associations between UTAUT-related variables and PA app use intention in Chinese university students. It is noted that body mass index (BMI) has been associated with PA app use in previous studies, so BMI may have an interaction effect with UTAUT-related variables [26]. The hypothesis that BMI would moderate the effects of UTAUT in explaining PA app use intention was also tested.

## Methods

### Study Design

This cross-sectional survey was conducted among university students in Guangzhou, China, from March 1, 2016, to April 20, 2016. A multistage stratified cluster sampling method was used. Selection criteria included all full-time students of universities located in Guangzhou admitted from 2013 to 2015 but excluded students whose majors were sports-related.

Universities in Guangzhou were divided into first and second class according to the Education Examinations Authority of Guangdong Province [31]. Two of the first-class universities and three of the second-class universities were selected using purposive sampling. Student majors from the universities were divided into five categories (natural science, agricultural science, medical science, humanities and social science, and engineering and technology science) according to China’s National Classification and Code Disciplines [32]. For each major category, we recruited at least 3 classes from each grade (students admitted in 2015, 2014, and 2013) separately. Contact persons were recruited from the selected universities. After training on study purpose, procedure, data collection, and quality control, they served as helpers to approach different classes and collect data. These contact persons distributed the questionnaires among their classmates, collected the answered questionnaires, and performed a preliminary verification of the quality of all answered questions. Financial reimbursement of 25 yuan (US $4) per hour was provided to them as compensation for their time.

### Theoretical Framework

**Figure 1:**
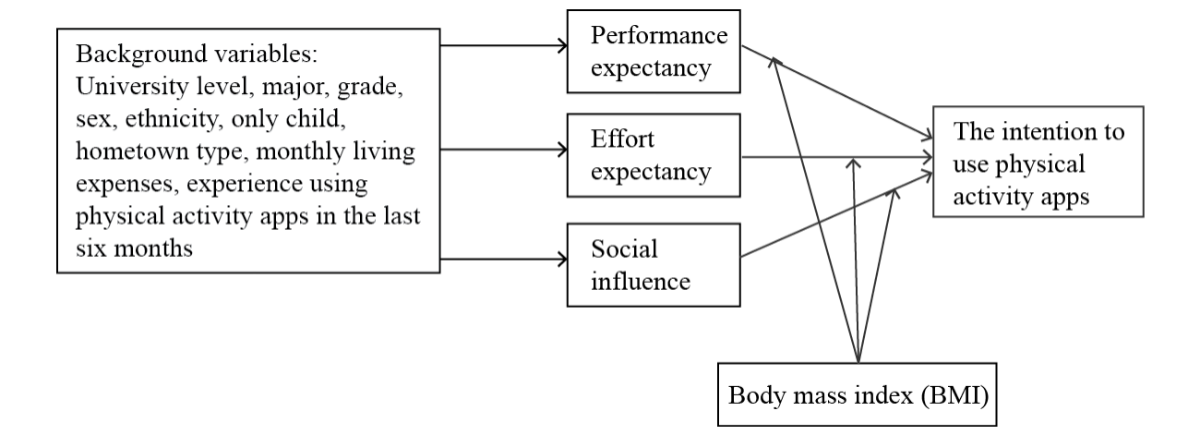
Modified model of unified theory of acceptance and use of technology.

According to UTAUT, a commonly used theory for identifying determinants of intention to use health technologies [29], direct determinants of intention are performance expectancy (PE, the degree to which using a technology will provide benefits in performing certain activities), effort expectancy (EE, the degree of ease associated with the use of the technology), and social influence (SI, the degree to which an individual perceives that important others believe he or she should use the system). Associations between PE, EE, SI, and intention are moderated by variables such as age and voluntariness of use. As PA app use is voluntary and age range does not vary much among university students, moderators were replaced by BMI (Figure 1).

### Data Collection

The questionnaire was anonymous and self-administered. Background variables including university, major, grade, sex, ethnicity, being the only child or not, hometown type, monthly living expenses (using 1000 yuan as a cutoff based on living expenses of university students in Guangzhou), and BMI were collected. BMI was calculated by self-reported height and weight, using 18.5 and 24 kg/m^2^ as cutoffs. BMI between 18.5 and 24 was defined as normal and BMI outside the range as abnormal (ie, BMI <18.5 means underweight and BMI ≥24 means overweight or obese, as defined by China’s Obesity Working Group). We measured intention to use PA apps with one question: Will you use PA apps in the coming 6 months? The experience of having used PA apps before was defined as having used PA apps at least once in the last 6 months. The questionnaire took about 5 to 8 minutes to finish.

PE, EE, and SI were measured with items generated by the research team according to literature and qualitative interviews. An item pool of questions used to measure UTAUT according to literature review was formed, and items were screened by the research group. We interviewed some university students to understand their thoughts on exercise app use according to UTAUT and collect their suggestions on the screened UTAUT items. A revised version of the UTAUT-related questionnaire measuring determinants PE, EE, and SI was developed after the research group discussion.

The determinants were assessed using multi-item measures scored by summing relevant item scores. Each item was scored on a 5-point Likert-type scale (1 = extremely disagree, 5 = extremely agree). Using a principal component analysis, factors were identified for the PE, EE, and SI scales, explaining 80.7%, 82.2%, and 76.3% of total variance, respectively. Cronbach alphas were 0.88, 0.89, and 0.68, respectively.

### Statistical Analyses

Univariate logistic regressions were performed to measure the associations between background variables, experience using PA apps in the last 6 months, and intention to use PA apps in the coming 6 months. Background variables with *P*<.10 in the univariate analyses were selected by a multivariate model using a stepwise method except for experience using PA apps in the last 6 months. Both univariate and multivariate analyses (adjusting for significant background variables and experience using PA apps in the last 6 months) were performed to calculate the association between the UTAUT construct and intention to use PA apps in the coming 6 months.

To identify the interaction effects of BMI on the associations between UTAUT constructs and intention to use PA apps, we performed multivariate logistic regression models, adjusting for significant background variables.

## Results

### Participant Characteristics

Among the students (from 55 classes) contacted, the response rate was 94.1% (1603/1704), and the effective response rate was 85.7% (1461/1704). Of all participants, 64.8% (947/1461) were from first-tier universities, 50.3% (735/1461) were male, 61.5% (899/1461) were not the only child, 48.9% (714/1461) were from a town or rural area, 67.8% (991/1461) had over 1000-yuan (US $150) monthly living expenses, and 33.3% (487/1461) had a BMI beyond the normal range (24.1%, 352/1461, were lower than the normal range while 9.2%, 135/1461, were overweight or obese; Table 1).

Of the participants, 41.8% (611/1461) intended to use PA apps. In univariate analysis, all background variables except for students’ grades and ethnicity were significantly associated with the intention to use PA apps (Table 1). In the multivariate analysis, students who were female (odds ratio [OR] 1.36, 95% CI 1.10-1.68) and from a capital city or municipality (OR 1.43, 95% CI 1.11-1.86) with monthly living expenses were over 1000 yuan (OR 1.44, 95% CI 1.14-1.83) and BMI beyond the normal range (OR 0.71, 95% CI 0.56-0.89) were more likely than others to intend to use PA apps (Table 1). Meanwhile, compared with those not having used PA apps in the last 6 months, participants having used apps in the last 6 months were more likely to intend to use PA apps in the coming 6 months (OR 4.16, *P*<.001; Table 1).

**Table 1:** Associations between background variables and the intention to use physical activity apps.

Characteristics		Statistical descriptive, n (%)	Intention to app physical activity apps				
			Yes, n (%)	OR_u_^a^	*P* value_u_	OR_m_^b^ (95% CI)	*P* value_m_
**University level**					**<.001**	**NS^c^**	**NS**
	Second-tier	514 (35.2)	181 (35.2)	1.00			
	First-tier	947 (64.8)	430 (45.4)	1.53			
**Major**						**NS**	**NS**
	Natural science	378 (25.9)	180 (47.6)	1.00	Ref		
	Agricultural science	257 (17.6)	100 (38.9)	0.70	.03		
	Medical science	241 (16.5)	95 (39.4)	0.72	.046		
	Humanities and social science	157 (10.7)	79 (50.3)	1.11	.57		
	Engineering and technology science	428 (29.3)	157 (36.7)	0.64	.002		
**Grade**						**—^d^**	**—**
	Freshman	532 (36.4)	230 (43.2)	1.00	Ref		
	Sophomore	513 (35.1)	211 (41.1)	0.92	.49		
	Junior	416 (28.5)	170 (40.9)	0.91	.46		
**Gender**					**.004**		**.005**
	Male	735 (50.3)	280 (38.1)	1.00		1.00	
	Female	726 (49.7)	331 (45.6)	1.36		1.36 (1.10-1.68)	
**Ethnicity**					**.75**	**—**	**—**
	Han	1369 (93.7)	574 (41.9)	1.00			
	Others	92 (6.3)	37 (40.2)	0.93			
**Only child**					**.75**	**NS**	**NS**
	No	899 (61.5)	356 (39.6)	1.00			
	Yes	562 (38.5)	255 (45.4)	1.27			
**Hometown type**							
	Town or rural area	714 (48.9)	267 (37.4)	1.00	Ref	1.00	Ref
	Noncapital city	377 (25.8)	168 (44.6)	1.35	.02	1.23 (0.95-1.60)	.11
	Capital city or municipality	370 (25.3)	176 (47.6)	1.52	.001	1.43 (1.11-1.86)	.007
**Monthly living expenses (yuan/month)**					**<.001**		**.002**
	≤1000	470 (32.2)	161 (34.3)	1.00		1.00	
	>1000	991 (67.8)	450 (45.4)	1.60		1.44 (1.14-1.83)	
**Body mass index**					**.006**		**.003**
	18.5-24 kg/m^2^	974 (66.7)	435 (44.7)	1.00		1.00	
	Beyond 18.5-24 kg/m^2^	487 (33.3)	176 (36.1)	0.73		0.71 (0.56-0.89)	
**Experience of using physical activity apps in the last 6 months**					**<.001**	**NA^e^**	**NA**
	No	924 (63.2)	271 (29.3)	1.00			
	Yes	537 (36.8)	340 (63.3)	4.16			

^a^Refers to univariate anlyses.

^b^Refers to multivariate analyses.

^c^NS: nonsignificant. Denotes variables with *P*<.10 in the univariate analyses that were not significant in the multivariate analyses.

^d^Denotes variables with *P*>.10 in the univariate analyses that were not used in the subsequent multivariate analyses.

^e^N/A: not applicable. Indicates that the experience of using physical activity apps in the last 6 months was not included in the multivariate analyses.

### Associations Between UTAUT-Related Variables and Intention to Use Physical Activity Apps

In the univariate analyses, all 3 UTAUT-related scales were significantly associated with intention to use PA apps (OR_u_ 1.22-1.49, *P*<.001). Such associations remained significant after adjusting for significant background variables (gender, hometown type, monthly living expenses, and BMI), and experience of using PA apps in the last 6 months (OR_a_ 1.10-1.31, *P*<.001).

In the associations between each UTAUT-related item and the studied outcome, all 8 items were significantly associated with the intention to use PA apps (OR_u_ 2.16-2.97; Table 2).

### Moderating Effects of Body Mass Index on the Associations Between UTAUT and Intention to Use Physical Activity Apps

**Figure 2:**
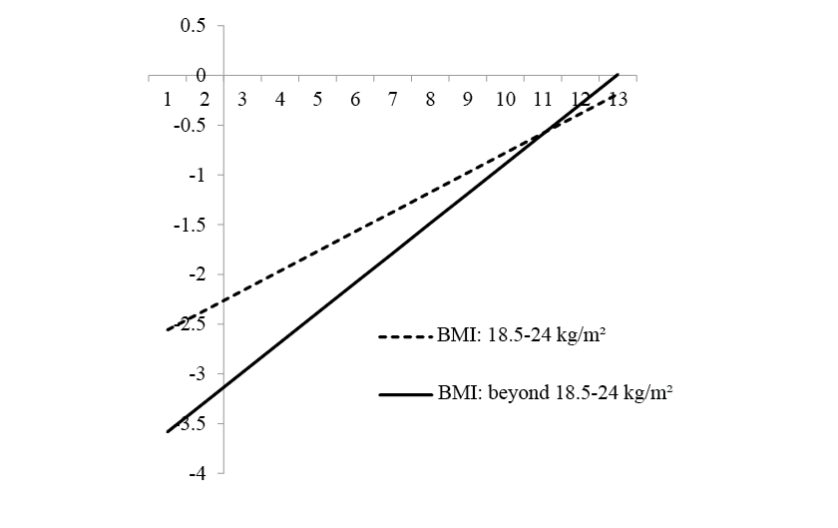
Interaction effect between body mass index and performance expectancy scale. BMI: body mass index.

One out of the three models considered presented statistically significant interaction effects: interaction between the BMI and the performance expectancy scale (beta 0.10, *P*<.001; Table 3). Higher scores on the PE scale (x-axis) were associated with higher log odds for intention to use PA apps (y-axis), but the strength of associations depended on the BMI, as seen by the slopes of the straight lines (Figure 2). The significant moderating effect indicated stronger associations between the PE scale and the intention to use PA apps among those whose BMI was beyond normal range (BMI≥24), as compared with those whose BMI was within normal range.

**Table 2:** Associations between UTAUT-related scales and the intention to use physical activity apps.

Scale		OR_u_^a^ (95% CI)	*P* value_u_	OR_a_^b^ (95% CI)	*P* value_a_
**Scale 1. Performance expectancy scale**		1.26 (1.20-1.32)	<.001	1.16 (1.11-1.22)	<.001
	Term 1.1. Using physical activity apps could inspire you to keep doing physical activity.	2.55 (2.06-3.16)			
	Term 1.2. Using physical activity apps could contribute to maintaining physical fitness.	2.60 (2.10-3.23)			
	Term 1.3. Using physical activity apps could contribute to maintaining good mental health.	2.16 (1.74-2.68)			
**Scale 2. Effort expectancy scale**		1.22 (1.16-1.27)	<.001	1.10 (1.04-1.15)	<.001
	Term 2.1. You can quickly master how to use physical activity apps.	2.57 (2.00-3.30)			
	Term 2.2. You can be proficient with using physical activity apps.	2.57 (2.04-3.24)			
	Term 2.3. Using physical activity apps is not difficult for you.	2.64 (2.05-3.41)			
**Scale 3. Social influence scale**		1.49 (1.39-1.60)	<.001	1.31 (1.21-1.42)	<.001
	Term 3.1. Your good friends are in favor of your using physical activity apps.	2.32 (1.88-2.87)			
	Term 3.2. Many of your friends are using physical activity apps.	2.97 (2.39-3.69)			

^a^Refers to univariate analyses.

^b^Refers to adjustment for gender, hometown type, monthly living expenses, experience of using physical activity apps in the last 6 months, and body mass index.

**Table 3:** Summary of logistic regression models testing significance of main and interaction effects of UTAUT-related scales and body mass index.

Model		Beta	SE (beta)	*P* value
**Model 1**				
	Performance expectancy scale	0.20	0.03	.05
	BMI^a^	–1.33	0.53	.01
	BMI × performance expectancy scale	0.10	0.05	<.001
**Model 2**				
	Effort expectancy scale	0.17	0.03	<.001
	BMI	–0.81	0.59	.17
	BMI × effort expectancy scale	0.04	0.05	.44
**Model 3**				
	Social influence scale	0.35	0.04	<.001
	BMI	–0.88	0.56	.12
	BMI × social influence scale	0.08	0.08	.29

^a^BMI: body mass index. BMI was divided into two levels: 0 = normal range of Chinese people (18.5-24 kg/m^2^) and 1 = beyond normal range.

## Discussion

### Principal Findings

To the best of our knowledge, this study is among the first to examine university students’ intention to use PA apps based on a new technology use–related model in China. Overall, university students showed a relatively high level (611/1461, 41.8%) of intention to use PA apps for increasing PA. Moreover, the number of participants having the intention to use PA apps in the coming 6 months was higher than the number of participants having used PA apps in the last 6 months (611 vs 537), which indicated that the intention to use PA apps of university students in Guangzhou showed an upward trend. This relatively strong intention suggests that future efforts to increase PA among university students with PA apps will be promising.

Of all the participants, 24.1% (352/1461) had a BMI lower than the normal range and 9.2% (135/1461) were overweight or obese according to their BMI. The rates of lower body weight and overweight or obese were both higher than the results of research conducted in Henan Province (low body weight: 14.7%; overweight or obese: 8.5%) [33] and northern Anhui Province (low body weight: 12.6%; overweight or obese: 8.3%) [34] among university students. But the rate of overweight or obesity in our study was a little lower than that found in one study conducted on university students in Zhejiang (low body weight: 8.2%; overweight or obese: 10.9%) [35]. Although there are regional differences in BMI, our results still indicated that the situation of university students’ BMI in Guangzhou being beyond the normal range was relatively serious. The physical health status of university students in Guangzhou is thus in urgent need of improvement.

In our study, PE, EE, and SI were all positively related to PA app use intention after adjusting for significant background variables. UTAUT can therefore be used to develop implementation interventions to increase the use of apps designed for improving PA among university students. SI showed a relatively strong association with the intention to use PA apps among the three scales with OR 1.49 (95% CI 1.39-1.60). This may be due to the community lifestyle of university students in China. University students live on campus, spend most of their time in class or in the dormitory, have close contact with their peers (classmates or roommates), and are easily influenced by their peers [36]. A survey conducted in European universities reached a similar conclusion that students’ alcohol use behavior was affected by their peers’ alcohol use behavior [37]. Social influence mainly refers to the influence from surrounding people and environment, which can explain why social influence plays a more important role in affecting the intention to use PA apps. To promote university students’ intention to use PA apps, schools or society may be able to achieve their goals with the aid of peer influence.

UTAUT-based interventions seem to be useful among all students regardless of their BMI. However, the strength of associations between UTAUT and PA app use intention may depend on other contextual factors. UTAUT-based interventions may have a better chance of success if they pay more attention to university students whose BMI are beyond the normal range. Such findings suggest that to improve physical activities among university students via app use, for students with BMI beyond normal range interventions enhancing PE (eg, peers sharing of benefits) could be considered, but for students with normal or low BMI intervention targeting PE alone may be less effective.

### Limitations

This study has some limitations. First, the observational design may not establish a causal relationship between independent variables and outcome. Although we collected data of use intention in the coming 6 months and use experience in the last 6 months, which guaranteed the time sequence, participants’ intention might still be based mainly on the time point of the survey. It was assumed that cognitive situations would be quite stable in the coming 6 months.

Second, although anonymity and privacy were guaranteed, a reporting bias due to social desirability and self-expectation may still exist. For example, as university students, the participants might give a high score to the EE scale. There were at least two items in each scale to try to avoid this problem, and Cronbach alpha was high for each scale.

Finally, we cannot assume that the results can be extrapolated widely without further research. A purposive sampling method was used to recruit participants, which may weaken the external validity of our sample. In addition, our study involved only one city and this city’s economic level is higher than most other cities in China, which might affect participants’ acceptability of new technology and health consciousness.

### Conclusions

This study, based on a theoretical approach to technology use, indicated which factors will need to be addressed to design an effective implementation intervention for the use of PA apps to increase PA among university students. Our findings indicated that university students’ intention to use PA apps was influenced by UTAUT-related constructs, but potential moderating effects of BMI should be kept in mind when UTAUT-based interventions are being developed. Different intervention strategies should be considered for students within and beyond the normal range of BMI.

## Abbreviations

BMI: body mass index
EE: effort expectancy
PA: physical activity
PE: performance expectancy
OR: odds ratio
SI: social influence
UTAUT: unified theory of acceptance and use of technology
